# Stem cell therapy combined with core decompression versus core decompression alone in the treatment of avascular necrosis of the femoral head: a systematic review and meta-analysis

**DOI:** 10.1186/s13018-023-04025-8

**Published:** 2023-08-02

**Authors:** Mengyuan Li, Duanyong Chen, Yuanchen Ma, Minghao Zheng, Qiujian Zheng

**Affiliations:** 1 Division of Joint Osteopathy and Traumatology, Guangdong Provincial People’s Hospital (Guangdong Academy of Medical Sciences), Southern Medical University, 106 Zhongshaner Road, Yuexiu District, Guangzhou, 510080 People’s Republic of China; 2 Centre for Orthopaedic Translational Research, School of Surgery, The University of Western Australia, M Block, QE2 Medical Centre, Monash Ave., Nedlands, WA 6009 Australia

**Keywords:** Avascular necrosis of femur head, Core decompression, Stem cells, Cell therapy

## Abstract

**Introduction:**

Accumulated clinical trials had been focused on stem cell therapy in combination of core decompression (CD) in the treatment of avascular necrosis of the femoral head (ANFH). Nonetheless, the results were inconclusive. Here, we performed a systematic review and meta-analysis of previous randomized controlled trials (RCTs) and retrospective studies to assess whether combined stem cell augmentation with CD improved the outcomes of ANFH compared with CD alone.

**Methods:**

The current study included 11 RCTs and 7 retrospective studies reporting the clinical outcomes of a total of 916 patients and 1257 hips. 557 and 700 hips received CD and CD plus stem cell therapy, respectively. To compare CD with CD plus stem cell therapy, we examined the clinical evaluating scores, the occurrence of the femoral head, radiologic progression and conversion to total hip arthroplasty (THA).

**Results:**

Only 10 studies reported significantly greater improvement in hip functions while combining stem cell procedure with CD. The pooled results in subgroup analysis indicated that stem cell group had a lower collapse rate on a mid-term basis (P = 0.001), when combined with mechanical support (P < 0.00001), and with extracted stem cells (P = 0.0002). Likewise, stem cell group had a lower radiographic progression rate at 2- to 5-year follow-up [*P* = 0.003], when combined with structural grafting (*P* < 0.00001), and with extracted stem cells (*P* = 0.004). Stem cell therapy resulted in an overall lower THA conversion rate (*P* < 0.0001) except that at a follow-up longer than 5 years.

**Conclusion:**

Stem cell therapy combined with core decompression was more effective in preventing collapse, radiographic progression and conversion to THA.

*Trial Registration *The current protocol has been registered in PROSPERO with the registration number: CRD42023417248.

**Supplementary Information:**

The online version contains supplementary material available at 10.1186/s13018-023-04025-8.

## Introduction

Avascular necrosis of the femoral head (ANFH), a prevalent disease for orthopedics especially in Asian population, was caused by impaired circulation of the femoral head [1]. The interruption of the blood supply leaded to structural rebuilding of the femoral head, then causes collapse of articular cartilage, and eventually gives rise to dysfunction and disability of hip [2]. The etiology of ANFH was multifactorial, including long-term use of glucocorticoids, alcohol abuse, and hip trauma [3]. In spite of good results of total hip arthroplasty (THA), it had been proved that the revision rate was up to 13.8% based on the recent registry data [4, 5]. Therefore, hip-preserving surgery had drawn much attention for the early stage of ANFH, including physical therapy, administration of bisphosphonates and/or nonsteroidal anti-inflammatory drugs, weight-bearing restrictions, multiple epiphyseal drilling augmented with autologous bone marrow implantation, free vascularized fibular grafts and osteotomies [6, 7]. Core decompression (CD) was the most frequently used among hip-preserving procedures, and the purpose was to reduce the intraosseous pressure and improve the blood supply of the femoral head.

Although CD has been utilized for more than 50 years, its efficacy was still controversial, this procedure did not demonstrate superior outcomes compared to other treatment modalities, thereby necessitating further research to determine the optimal treatment approach for ANFH [8]. Steinberg et al. reported that average of 36% of patients after CD would ultimately receive THA [9]. CD alone may not be effective in improving the pain and function on a long-term basis, especially for the cases of mid-stages (Association Research Circulation Osseous, ARCO stage II/III) [10]. Recent research suggested that poor prognosis of the CD was associated with male gender, longer duration of symptoms prior to treatment, higher visual analogue scale (VAS) scores and lower Harris Hip Scores (HHS) [11]. In 1997, Hernigou el al. proposed the application of bone marrow cells for the treatment of ANFH [12]. In the past two decades, the literature had revealed that the pathogenesis of ANFH was strongly related to the decreased pool of osteoprogenitor cells in the bone marrow of the femoral head [12, 13]. It was well-known that stem cells had capacity of multipotent differentiation and could differentiate into osteoprogenitor cells, vascular progenitor cells, chondroblasts and osteoblasts, etc., improving revascularization and promoting the reconstruction of the bone tissue in femoral head. Based on this phenomenon, mesenchymal stem cells (MSCs) transplantation to the necrotic area was considered to be an effective treatment for early-stage cases.

Up till nowadays, several authors had published systematic reviews about CD plus stem cells therapy for ANFH [14–18]. Nonetheless, these previous studies included several limitations. First of all, the type and number of stem cells were not consistent in those studies. Secondly, although the incidence of collapse was proved to be a critical outcome, few studies have synthesized and assessed this parameter. Additionally, with the publishment of the long-term results of stem cells therapy, we believed that it is necessary to update the systematic review and meta-analysis in this field. The aim of the present systematic review and meta-analysis was to evaluate whether CD combined with stem cells therapy in the early stage of ANFH can reduce pain, improve hip function and prevent collapse of the head. The hypothesis was that: (1) the augmentation using cell therapy would postpone the progression of ANFH and reduce the conversion rate of THA. (2) The mechanical support of the subchondral bone in the femoral head would be advantageous. (3) The outcomes would not vary while using either MSCs or bone marrow aspirate concentrate (BMAC).

## Methods

### Protocol and registration

The current protocol has been registered in PROSPERO with the registration number: CRD42023417248, following PRISMA guidelines [19].

### Eligibility criteria

The studies included in our present meta-analysis were in strict accordance with PICOS criteria as follows: patients (P): the patients were older than 18 years age and diagnosed with ANFH; intervention (I): the treatments were based on core decompression and mechanical supporting procedures, and various stem cells including peripheral blood mesenchymal stem cells, bone marrow stem cells, bone marrow aspirate concentrates, bone marrow mononuclear cells, etc., were added to the surgical site; Comparison (C): core decompression with mechanical supporting procedures without stem cells therapy was as direct comparison; Outcomes(O): the primary outcomes were the rate of conversion to THA and the rate of radiographic collapse after intervention; the secondary outcomes were diverse post-operative clinical evaluating scores including HHS, Western Ontario and McMaster Universities Osteoarthritis Index (WOMAC) and VAS, etc. All trials we included were controlled trials.

The exclusion criteria were (1) duplicated studies, animal or cadaver studies, biomechanical studies, reviews, correspondence or technical notes; (2) the hip of patients has received previous surgery; (3) uncontrolled trials; (4) biological augmentation interventions used by study group were without stem cells.

### Search strategy

The literature was searched using the following databases: PubMed, EMBASE, Web of Science databases, and the Cochrane Central Register of Controlled Trials for reports published from their commencement to March 2023 to identify the case-controlled studies, cohort studies, prospective studies and randomized controlled trials (RCT) that have compared the effects of CD with or without stem cells in the treatment of ANFH. The key term strings were used as follows: “osteonecrosis”, “avascular necrosis”, “femur head”, “stem cells”, “progenitor cells”, “cell therapy”, “core decompression”, “bone graft”. A search of the references on recent meta-analyses and reports of meetings was also undertaken. The language was restricted to English. Eligible studies were selected by screening the title or abstract. If this was deemed insufficient, the entire article was reviewed.

### Selection and data collection

Two independent reviewers (MYL, DYC) followed a standardized form to extract data from articles without filters or constraints in the database search and independently assessed all the titles and abstracts for eligibility. The full text was obtained if at least one author judged a study to be eligible. Disagreements were resolved by consensus.

### Data items

The extracted data elements included authors, publication date, evidence-based level, population, number of participants and hips, ratio of gender, mean follow-up, mean age, etiology, stage of necrosis (Ficat/ARCO), type of mechanical support after core decompression, type and number of stem cells. The number and rate of conversion to THA and radiographic collapse after intervention were recorded as primary outcome. The clinical functional scores of the hip, including HHS, WOMAC, VAS, were extracted as secondary outcomes. The post-operative data were based on the last time-point of follow-up because of the diverse follow-up time in the included studies.

### Assessment of the risk of bias

Following the flowchart of the Cochrane Handbook for Systematic Reviews of Interventions [20], the reviewers (QJZ, YCM) independently assessed the random sequence generation, allocation concealment, blinding of participants and personnel, blinded evaluation of the outcome, the completeness of the outcome data, selective reporting, and other bias. Each of the domains was scored as “no risk of bias”, “high risk of bias”, or “unclear”. The Newcastle–Ottawa scale was introduced to assess the methodological quality of the included retrospective studies [21]. The scoring system included the representative of the study, exposure ascertainment, comparability of simultaneous group, assessment, follow-up, possible risk in selection bias and missing data. A score of 0–9 was allocated to each non-RCT, and the study scored higher than 6 was considered to be of high quality.

### Assessment of the quality of recommendations

The quality of the evidence was evaluated based on the evidence profile using the GRADE (Grading of Recommendations Assessment, Development and Evaluation) system [22]. This approach enables a rating of the overall quality based on the evidence for risk of bias, publication bias, imprecision, inconsistency, and indirectness. The quality of evidence can be classified as very low-, low-, moderate-, or high-quality. The evidence quality was graded using the GRADE profile software (GRADEpro 3.6).

### Data synthesis and analysis

Review Manager (RevMan 5.3, The Cochrane Collaboration, Copenhagen, Denmark) was used to extract data for statistical analysis. Chi-square test was used for heterogeneity testing if the research object, intervention measures and method of assessing outcome were identical. Mantel–Haenszel test (M–H) was used for enumeration data, and inverse variance (IV) was used for measurement data. The inspection was largely supported by the *I*^2^ index, which quantifies the proportion of variability in outcomes attributable to heterogeneity rather than chance across various trials. When I^2^ was less than 50%, it indicated that the heterogeneity among different studies was small, and a fixed-effects model can be used for statistical analysis. However, when I^2^ was greater than 50%, it indicated that the heterogeneity among different studies was large, and a random-effects model should be used for statistical analysis. The odds ratio (OR) was calculated for enumeration data; 95% confidence intervals (CIs) were also calculated for all meta-analyses (*P* < 0.05). The presence of publication bias of the primary outcome was tested by Egger test and illustrated as Funnel plot using STATA (STATA 17.0, The StataCorp, Texas, USA), P < 0.05 indicated significant publication bias.

## Results

### Search results

**Fig. 1:**
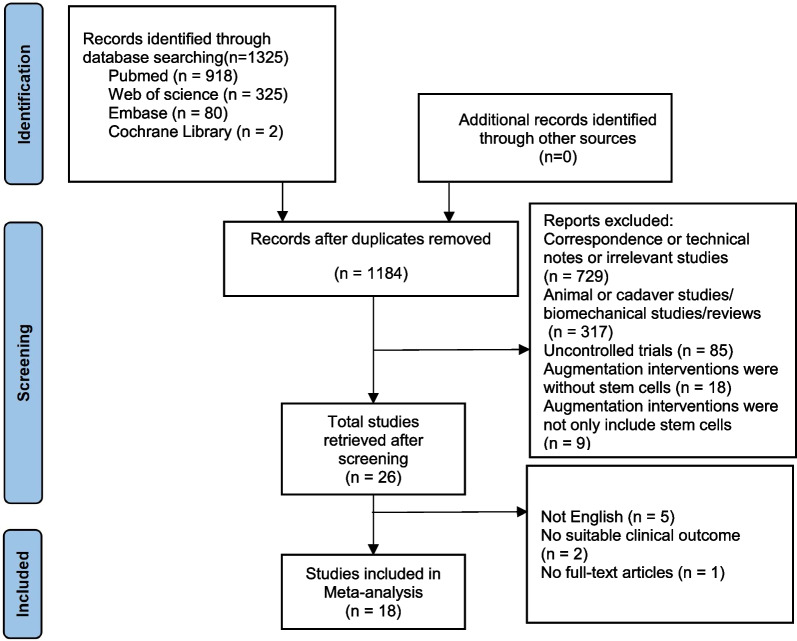
Flowchart of the selection of the relevant clinical studies

A total of 1325 articles were identified from the databases. A total of 141 studies were removed for duplication, and then, 729 studies were screened because they were correspondence or technical notes or irrelevant studies. A total of 317 studies were excluded because they were based on animal models or cadaver species, biomechanical studies and reviews. A further 85 non-controlled trials were also excluded. Twenty-seven trials were excluded from the remaining because their augmented interventions were not stem cells or not only stem cells. Of the remaining 26 studies, five were not in English, two did not have suitable clinical outcome, and one was not available in full-text articles, and therefore, they were also excluded. After the application of exclusion criteria, a total of 18 papers, all in English, were included in this meta-analysis (Fig. 1) [10, 23–39]. All selected studies used a conventional parallel group design, comparing CD versus CD plus stem cell therapy. Of the 18 identified studies, 11 studies were randomized controlled design [10, 28–34, 37–39], and the other 7 were retrospective studies [23–27, 35, 36].

### Assessment of the risk of bias

**Fig. 2:**
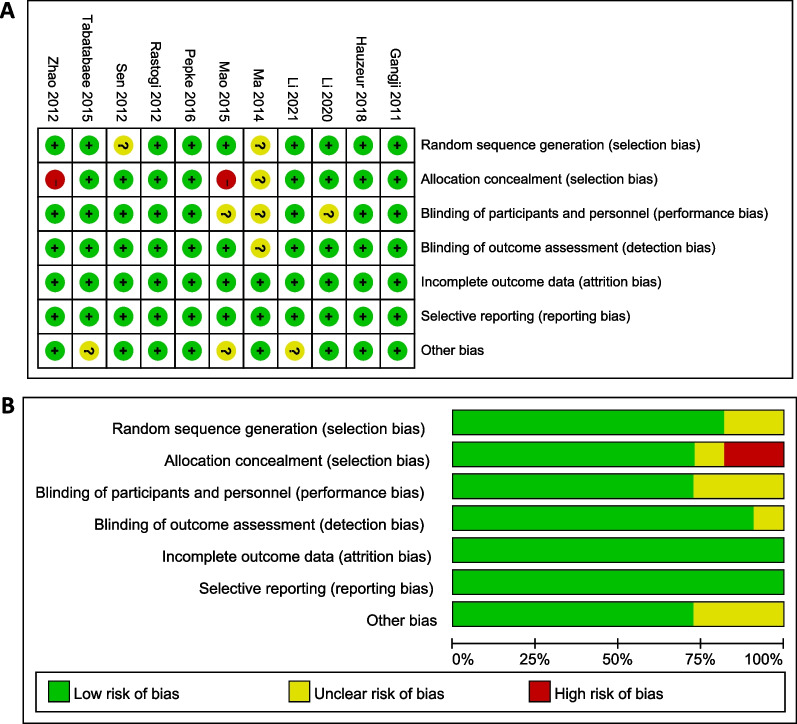
Graph showing risk of bias assessment. Low risk: + ; high risk: –; unclear: ?

Figure 2A, B illustrated the quality of each RCT. Table 1 indicated the quality of the 7 included retrospective studies.

**Table 1:** Risk of bias in the observational clinical studies using Ottawa–Newcastle Scale

Study	Selection				Comparability	Exposure			Quality score
	Adequate definition of cases	Representativeness of the cases	Selection of controls	Definition of controls	Control for important factor	Ascertainment of exposure	Same method of ascertainment for cases and controls	Non-response rate	
Yamasaki (2010)	★	★	–	★	★	–	★	★	6
Liu (2013)	★	★	–	★	★★	★	★	★	8
Lim (2013)	★	–	–	★	★	★	★	★	6
Pilge (2016)	★	★	★	★	★★	★	★	★	9
Cruz-Pardos (2016)	★	★	★	★	★	★	★	★	8
Hernigou (2018)	★	★	★	★	★★	★	★	–	8
Kang (2018)	★	★	★	★	★★	★	★	★	9

### Study characteristics

Seventeen of the included studies evaluated the conversion of hips to THA [10, 23–27, 29–39]. Eleven studies evaluated the radiographic progression [10, 23, 24, 26, 29–34, 39]. Nine studies recorded the radiographic progression [10, 23, 26, 29–33, 39]. With the definition of collapse as the femoral head as it progressing to ARCO stage III/Ficat stage IIB/Steinburg stage IV/Japanese Orthopaedic Association (JOA) stage II or subchondral fracture, eight studies evaluated the radiographic progression [10, 23, 24, 26, 32, 33, 35, 36].

#### Demographic, staging and etiologies matching

Table 2 provided the demographic, intervention and baseline data. A total of 905 patients and 1257 hips were included in the systematic review and meta-analysis. The age of the patients enrolled in these included studies ranging from 31 to 49.7. 556 and 701 hips received CD and CD plus stem cell therapy, respectively. The included studies had a minimum follow-up of 2 years. The study of the longest follow-up came from France, where Hernigou [35] reported a 25-year-result. Four kinds of staging systems were utilized in all of the studies. Among them, 11 studies [10, 24–26, 28, 30–34, 39] followed ARCO classification, 5 studies[27, 29, 36–38] reported data on the base of Ficat classification, and the left 2 studies used JOA [23] or Steinberg staging system [35]. Seventeen studies [10, 23–33, 35–39] documented and matched the etiologies of ANFH in their cases. Two well-known risk factors, Corticosteroid use and alcohol-abuse, took up 45% and 21% as the cause. Nonetheless, the etiology in a high proportion of patients (25%) was still ambiguous and thus, reported as idiopathic (Table 2).

**Table 2:** Demographic and descriptive characteristics of the included studies

Author	Country	LoE	Patients (N)		Hip (N)		Age (ys)		Sex (M:F)		Stage		Etiology (N)	Technique of CD	Mechanical support	Type and Number of Stem Cells	Follow-up (years)
			SG	CG	SG	CG	SG	CG	SG	CG	SG	CG					
											JOA						
Yamasaki (2010)	Japan	III	22	8	30	9	41	49	14:8	7:1	I:II:III 2:25:3	I:II:III 30:8:0	SG: corticosteroids (22): Alcohol-abuse (6): Idiopathic (2); CG: corticosteroids (2): Alcohol-abuse (4): Idiopathic (3)	Single drilling with a 6 ~ 10 mm bur	Interconnected porous calcium hydroxyapatite	BMMSCs 40 ml 1 × 10^9^	2.5
											ARCO						
Gangji (2011)	Belgium	I	10	9	13	11	42.5	45.7	Total: 10:9		I:II 2:11	I:II 2:9	SG: Corticosteroids (11): Alcohol-abuse (1): Idiopathic (1); CG: Corticosteroids (9): Alcohol-abuse (1): Idiopathic (1)	Drilling with a 3 mm trephine	No mechanical support	BMAC 50 ml 1.9 × 10^9^	5
Zhao (2012)	China	I	50	50	53	51	32.7	33.8	27:23	26:24	I:II 3:50	I:II 2:49	SG: Corticosteroids (11): Alcohol-abuse (11): Idiopathic (17): Trauma (8): Caisson disease (6); CG: corticosteroids (13): Alcohol-abuse (8): Idiopathic (13): Trauma (12): Caisson disease (5)	Single drilling with 10 mm trephine	No mechanical support	BMMSCs 2 ml 2 × 10^6^	5
Rastogi (2012)	India	I	40		30	30	34.7	33.0	5:2	3:1	I, II, III		SG: Corticosteroids (10): Alcohol-abuse (2): Idiopathic (14): Other (4); CG: Corticosteroids (8): Alcohol-abuse (6): Idiopathic (12): Other (4)	Reaming with a 4.5 mm cannulated reamer	No mechanical support	BMMCs 5 ml 0.1 × 10^9^	2
Sen (2012)	India	I	26	25	26	25	NA		19:7	18:7	I, II		Total: Corticosteroids (20): Alcohol-abuse (8): Idiopathic (2): Trauma (17): Pregnancy (2): Cushing disease (2)	Triple drilling with 4 mm diameter	No mechanical support	BMAC 2 ml 5 × 10^8^	2
Liu (2013)	China	III	17	17	28	27	38.0	38.1	13:4	14:3	IIb:IIc 13:15	IIb:IIc 12:15	SG: Corticosteroids (10): Alcohol-abuse (15): Idiopathic (3); CG: Corticosteroids (9): Alcohol-abuse (14): Idiopathic (4)	Single drilling with a 10 mm outer diameter	Granular porous medical nano-hydroxyapatite / polyamide 66 composite bone filling material	BMMCs 5 ml 0.16 × 10^9^	2.2
Tabatabaee (2015)	Iran	I	18		14	14	31.0	26.8	9:5	10:4	I:II:III 3:9:2	I:II:III 2:7:5	SG: Corticosteroids (10): Idiopathic (4); CG: Corticosteroids (9): Idiopathic (5)	Single drilling with 2.7 mm trephine	Allograft bone	BMAC 58 ml 0.5 × 10^9^	2
Mao (2015)	China	I	30	25	48	41	34.6	36.1	17:13	13:12	I:II:III 8:29:11	I:II:III 10:23:8	SG: corticosteroids (16); alcohol-abuse (18): idiopathic (14); CG: corticosteroids (15): Alcohol-abuse (14): Idiopathic (12)	Single drilling with 14 mm trephine	Porous tantalum rod	PBSCs 60 ml 0.25 × 10^9^	3
Pilge (2016)	Germany	III	10	10	10	10	38.4	38.3	9:1	8:2	II:III:IV 6:3:1	II:III:IV 6:3:1	SG: Corticosteroids (4): Idiopathic (4): Other (2); CG: Corticosteroids (2): Idiopathic (5): Other (3);	NA	No mechanical support	BMAC 7-10 ml	2.5
Pepke (2016)	Germany	I	24		11	14	44.3	44.5	10:1	12:2	II 11	II 14	NA	Triple drilling with 2 mm Kirschner wire (K-wire)	No mechanical support	BMAC 10 ml 1.19 × 10^9^	2
Kang (2018)	Korea	III	50	50	53	53	46.0	47.3	36:14	38:12	I:II:III:IV 1:29:19:4	I:II:III:IV 1:29:19:4	SG: Corticosteroids (5): Alcohol-abuse (19): Idiopathic (24): Other (5); CG: corticosteroids (5): alcohol-abuse (19): idiopathic (24): other (5)	Multiple drilling with 2 mm K-wires	No mechanical support	BMMSCs 15 ml 0.2 × 10^9^	4.3
Hauzeur (2018)	Belgium	I	19	19	23	23	48.0	49.7	14:5	13:6	III 23	III 23	SG: corticosteroids (12): alcohol-abuse (8): idiopathic (1): drepanocytosis (2); CG: corticosteroids (13): alcohol-abuse (7): idiopathic (3)	Drilling with a 4 mm trephine	No mechanical support	BMAC 50 ml 3.46 × 10^9^	2
											Ficat						
Lim (2013)	Korea	III	86	21	128	31	36.3	34.4	69:17	16:5	IIa:IIb:III 42:37:49	IIa:IIb:III 14:9:8	SG: corticosteroids (48): alcohol-abuse (20): idiopathic (15): other (3); CG: corticosteroids (6): alcohol-abuse (4): idiopathic (10): other (1)	Single drilling with 3 mm trephine	No mechanical support	BMAC 30 ml 8.7 × 10^8^	7.3
Ma (2014)	China	I	21	18	25	24	35.6	34.8	15:6	13:5	I:II:III 3:17:5	I:II:III 4:15:5	SG: corticosteroids (13): alcohol-abuse (4): idiopathic (6); CG: corticosteroids (13): alcohol-abuse (3): idiopathic (6)	Single drilling with 10 mm trephine	Autologous bone graft	BMAC 1 ml 3 × 10^9^	2
Cruz-Pardos (2016)	Spain	III	45		41	19	42.6	36.7	27:14	14:5	I:II 8:33	I:II 5:11	SG: corticosteroids (14): alcohol-abuse (3): idiopathic (12): other (8); CG: Corticosteroids (6): alcohol-abuse (5): idiopathic (6): other (2)	Single drilling with 4 mm trephine	No mechanical support	BMAC 20 ml	3.8
Li (2020)	China	I	17	14	21	20	34.1	38.2	12:5	10:4	II:III 11:10	II:III 11:9	SG: corticosteroids (10): alcohol-abuse (6): idiopathic (5); CG: corticosteroids (9): alcohol-abuse (5): idiopathic (6)	Single drilling with 10 mm trephine	Autologous bone graft	BMAC 1 ml 3 × 10^9^	10
Li (2021)	China	I	17	23	22	29	39.4	35.4	12:5	19:4	I:II:III:IV 1:19:2:0	I:II:III:IV 1:20:6:2	SG: corticosteroids (8): alcohol-abuse (5): idiopathic (9); CG: corticosteroids (3): alcohol-abuse (9): idiopathic (17)	Single drilling with 10 mm trephine	Autologous bone graft and angioconductive bioceramic rod	BMAC 3 ml	2
											Steinberg						
Hernigou (2018)	France	II	125	125	125	125	total: 36		78:47	78:47	I:II 69:56		Corticosteroids (125)	Single drilling with 4 mm trocard	No mechanical support	BMAC 90,000 ± 25,000	25
Total and weighted averages	Asia (12/18), Europe (6/18)	I (11/18), II (1/18), III (6/18)	916		1257		/		392:181	312:145	/		Corticosteroids (45%), alcohol-abuse (21%), Idiopathic (25%), Other (9%) (extracted from the available data)	Single drilling (14/18), multiple drilling (3/18), NA (1/18)	Mechanical support (7/18), no mechanical support (11/18)	BMAC (12/18), BMMSCs (3/18), BMMCs (2/18), PBSCs(1/18)	7.5

ARCO Association Research Circulation Osseous, BMAC bone marrow aspirate concentrate, BMMCs bone marrow mononuclear cells, BMMSCs bone marrow mesenchymal stem cells, CD core decompression, CG control group, JOA Japanese Orthopaedic Association, LoE level of evidence, PBSCs peripheral blood stem cells, SG stem cell group

##### Surgical technique

CD was the fundamental procedure in all included studies. Nonetheless, the technique had discrepancies. Fourteen studies applied single drilling technique, whereas 3 studies chose multiple drilling. Kirschner wires [26, 34], burs [23], reamers [39], trephines [10, 27, 29–33, 36–38] and trocards [35] were utilized as the tools for decompression. The diameters of the tunnels were also diverse, ranging from 2 to 14 mm (Table 2).

Mechanical structural augmentations were described in 7 studies [23, 24, 29, 32, 33, 37, 38], 3 of which used autologous bone graft [29, 37], and Li [38] used auto bone-grafting plus angio-conductive bio-ceramic rod. Interconnected porous calcium hydroxyapatite [23], Granular porous medical nano-hydroxyapatite, polyamide 66 composite bone filling material [24], porous tantalum rod [33] and allograft bone [32] were also used for structural support. In the other 18 studies, no specific mechanical augmentation was employed (Table 2).

##### Type of stem cell therapy

All the included studied performed 4 kinds of cell therapy. Twelve studies [10, 25, 27–30, 32, 34–38] applied bone marrow aspirate concentrate (BMAC) in their cohorts. Three [23, 26, 31], two [24, 39] and one [33] used bone marrow mesenchymal stem cells (BMMSCs), bone marrow mononuclear cells (BMMCs) and peripheral blood stem cells (PBSCs) as biologic augmentation, respectively. There was a heterogeneity when concerning the number of cells. In the studies utilizing BMAC, the number ranged from 90,000 to 3.46 × 10^9^. As for those applying stem cell direct injection, the number of cells ranged from 2 × 10^6^ to 0.25 × 10^9^ (Table 2).

##### Clinical outcome

The most frequently used clinical scoring system among all the included studies was Harris Hip Score (HHS) [24, 28, 33–35, 38, 39] and visual analogue scale (VAS) [10, 24, 26, 29, 30, 32, 34, 35, 37]. To add to that, 6 studies [10, 29, 30, 32, 35, 37] assessed the clinical function based on Western Ontario and McMaster Universities Arthritis Index osteoarthritis scoring (WOMAC). Three [29, 30, 37] and two [25, 36] studies reported Laseque index and Merle D’Aubigné and Postel score, respectively. However, since some data did not obey normal distribution and thus, was not reported in the form of “average ± standard difference” [10, 23, 25, 26, 36, 37, 39]. Some studies reported the scores in the figure or did not report the exact value [27, 30, 31, 34]. These reasons resulted in the difficulty to extract the data to synthesize a forest plot.

In 16 included studies, the pre-operative functional scores were matched between stem cell and control group despite of diverse scoring system. Two studies did not compare the post-operative clinical scores between groups [27, 31]. Ten studies reported significantly greater improvement in hip functions while combining stem cell procedure to CD despite of using different scoring systems and the diverse follow-up duration [24, 25, 28, 30–33, 35, 37, 38]. Nonetheless, the remaining 6 studies did not detect statistically significant differences in clinical scores between the two treatment groups [10, 26, 29, 34, 36, 39] (Table 3).

**Table 3:** Clinical scores before and after surgery

Author	Scoring system	Pre-operative score		Post-operative score		Comparison between SG and CG
		SG	CG	SG	CG	
Yamasaki, 2010	Pain score	14.7 (13 to 16)	15.2 (14 to 17)	17.0 (15 to 18)	14.2 (12 to 15)	No direct statistical comparison was performed
Gangji, 2011	VAS Laseque index WOMAC	32.8 ± 7.1 7.2 ± 1.2 NA	46 ± 7.2 8.6 ± 1.4 NA	20.8 ± 7.7 4.8 ± 1.8 NA	49.8 8.7 NA	SG showed improved VAS at 36 months and improved Laseque index in comparison with CG, while no improvement was indicated in WOMAC
Zhao, 2012	HHS	NA	NA	NA	NA	The mean HHS in hips of ARCO stage IC, IIA, IIB, IIC in SG were higher, and the percent increase in hips of ARCO stage IIB and IIC were greater in SG
Sen, 2012	HHS	66.2 ± 13.0	65.7 ± 15.2	82.4 ± 9.2	77.4 ± 17.0	SG had a higher HHS and its domains than CG
Rastogi, 2013	HHS	46.8	47.1	78.6	66.8	There was no statistically significant difference between SG and CG in the degree of change of HHS
Liu, 2013	VAS HHS	63.6 ± 9.5 63.6 ± 2.6	62.6 ± 6.6 64.6 ± 2.9	21.4 ± 9.4 81.8 ± 2.6	30.2 ± 6.4 76.5 ± 2.9	The magnitude of improvement in HHS and VAS were greater in SG
Lim, 2013	HHS	NA	NA	NA	NA	No direct statistical comparison in scores between was performed
Ma, 2014	VAS WOMAC Laseque index	35.6 ± 4.2 27.8 ± 4.2 9.6 ± 1.0	35.2 ± 3.4 24.8 9.8	16.9 ± 3.6 14.8 ± 3.0 5.8 ± 0.9	26.5 ± 2.6 21.5 7.0	No statistical differences were detected in all clinical scores
Tabatabaee, 2015	VAS WOMAC	35.9 ± 4.5 32.0 ± 3.8	38.6 ± 4.6 35.9 ± 2.7	16.0 ± 3.7 9.7 ± 1.8	32.1 ± 4.1 27.2 ± 3.7	VAS and WOMAC were significantly lower in SG
Mao, 2015	HHS	62.7 ± 11.1	64.6 ± 8.6	88.1 ± 3.3	78.5 ± 8.7	SG had higher improvement with regard of HHS compared with CG
Pilge, 2016	MAP	13.5	14.3	15.2	14.1	MAP hip score improved post-operatively in SG but not in CG
Cruz-Pardos, 2016	MAP	13.6	14.1	14.9 ± 2.7	14.4 ± 2.8	Similar MAP hip score in both groups post-operatively
Pepke, 2016	VAS HHS	4.8 60.8	5.7 62.2	2.3 81.8	2.8 77.0	No significant differences were detected in VAS or HHS post-operatively
Hernigou, 2018	VAS HHS WOMAC	40.5 ± 5.2 76 (65 to 82) 40 ± 4.6	41.2 ± 6.5 87.3 (80 to 90) 38 ± 5.2	1 year:12.0 ± 3.5 2 years:94 (85 to 100) 25 years:8.6 ± 2.3	1 year:27.0 ± 4.4 2 years:80.2(70 to 85) 25 years:12.5 ± 2.3	SG had better reduction in VAS and HHS in SG as compared with CG within 1 year post-operatively. However, No direct statistical comparison between groups in scores was performed with respect to long-term follow-up
Kang, 2018	VAS	48.0 ± 13.0	42.0 ± 11.0	23.0	21.0	No significant differences were detected in VAS
Hauzeur, 2018	VAS WOMAC	58.4 ± 4.5 10.9	46.7 ± 5.7 10.9	–7.7 ± 5.9 7.9	–2.3 ± 6.4 10.2	No significant differences were detected in VAS or WOMAC
Li, 2020	VAS WOMAC Laseque index	40.0 (20 to 100) 21.0 (2 to 80) 9.0 (1 to 21)	45.0 (20 to 100) 33 (8 to 91) 10.0 (3 to 20)	10 (0 to 50) 8.0 (1 to 31) 4.0 (0 to 12)	35 (10 to 70) 32.5 (2 to 72) 9.0 (0 to 18)	VAS, Laseque index and WOMAC were overall better in SG than that in CG
Li, 2021	HHS	67.2 ± 9.2	68.5 ± 13.1	84.1 ± 14.2	72.8 ± 24.1	SG had a higher HHS than CG, especially in HHS-Function Scores

ARCO Association Research Circulation Osseous, CG control group, HHS Harris hip score, MAP Merle D’Aubigné and Postel score, VAS visual analogue scale, WOMAC Western Ontario and McMaster Universities Arthritis Index osteoarthritis scoring, SG stem cell group

##### Synthesis of results

###### Collapse of the femoral head

Based on the current literature, whether the femoral head collapsing had been considered as a critical prognostic factor of hip-preserving procedure. Among the included studies, 5 [23, 24, 33, 35, 36] compared the occurrence of collapse directly, and 3 [10, 26, 32] evaluated collapses based on the classification system. We extracted the data from these 8 [10, 23, 24, 26, 32, 33, 35, 36] studies. Additionally, we divided the 8 studies into subgroups according the duration of follow-up, whether the structural support was employed and the type of stem cell therapy and conducted a meta-analysis of these subgroups.

**Fig. 3:**
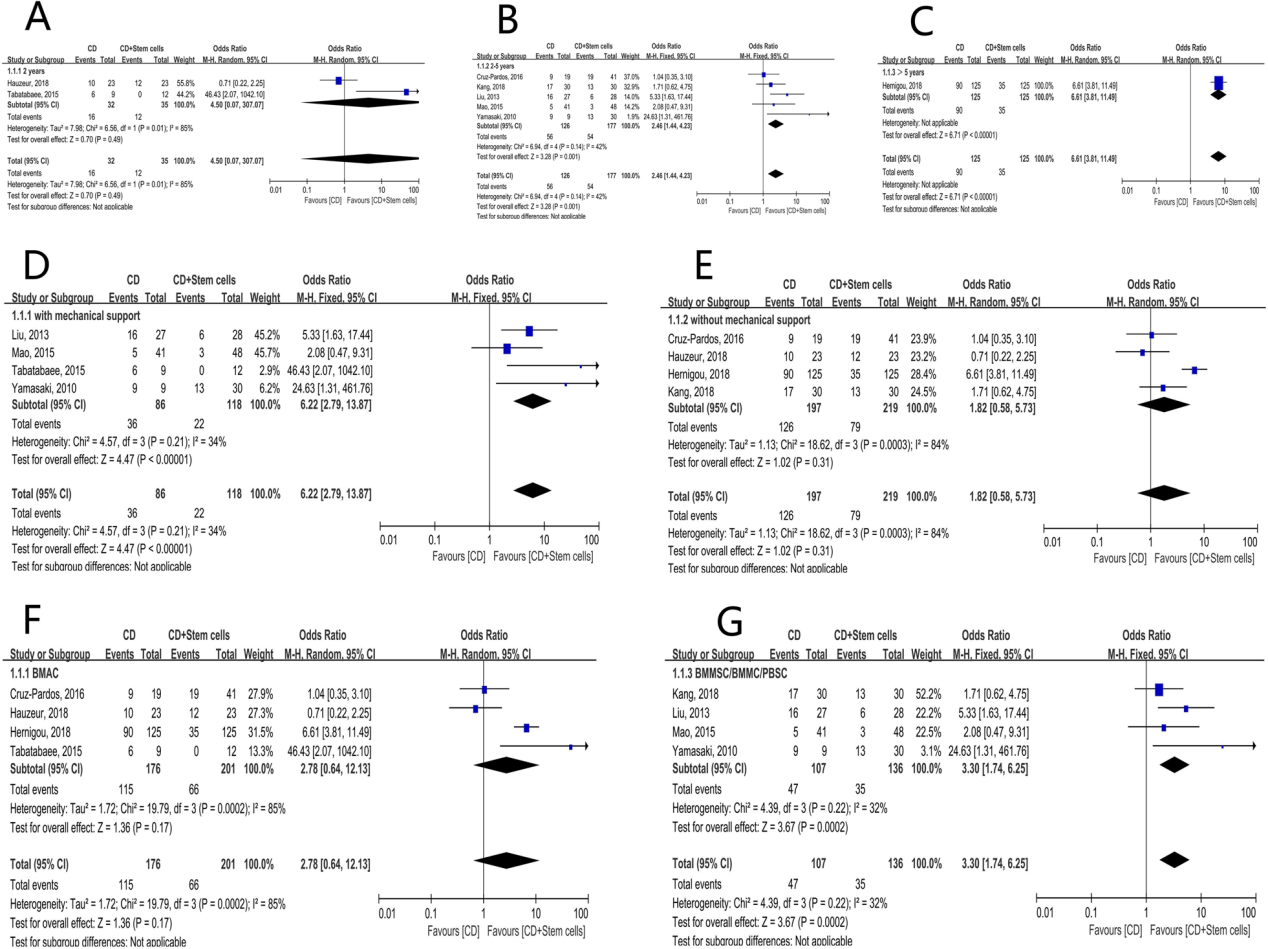
Forest plots of the rate of collapse. Subgroup analysis according to **A** the duration of follow-up in 2 years, **B** the duration of follow-up in 2–5 years, **C** the duration of follow-up longer than 5 years, **D** with structural support, **E** without structural support, **F** stem cell therapy of the BMAC group, **G** stem cell therapy of the BMMSCs/BMMSs/PBSCs group. (BMAC bone marrow aspirate concentrate, BMMCs bone marrow mononuclear cells, BMMSCs bone marrow mesenchymal stem cells, CD core decompression, CI confidence interval, df degree of freedom, M-H Mantel–Haenszel test, PBSCs peripheral blood stem cells)

The 8 studies were firstly categorized based on the duration of follow-up: (1) 2 year-follow-up: 12/35 (34.2%) hips in the stem cell group and 16/32 (50.0%) in the control group were observed with collapse of the femoral head. It was not statistically significant [OR = 4.50; 95% CI (0.07, 307.07); *P* = 0.49] (Fig. 3A). (2) 2- to 5 year-follow-up: 54/177 (30.5%) hips in the stem cell group and 56/126 (44.4%) in the control group were observed with collapse of the femoral head. It was statistically significant [OR = 2.46; 95% CI (1.43, 4.23); *P* = 0.001] (Fig. 3B). (3) longer than 5 year-follow-up: only 1 study was in this subgroup, collapses of the femoral heads were observed in 35/125 (28.0%) hips in the stem cell group and 90/125 (72.0%) in the control group (Fig. 3C).

The included studies were then divided into CD with and without mechanical support subgroup: (1) CD with mechanical support: (18.6%) (22/118) of femoral heads in the stem cell group and (36/86) (41.9%) in the control group collapsed. It was statistically significant [OR = 6.22; 95% CI (2.79, 13.87); *P* < 0.00001] (Fig. 3D). (2) CD without mechanical support (79/219) hips (36.1%) in the stem cell group and (126/197) (64.0%) in the control group were observed with collapse of the femoral head. It was not statistically significant [OR = 1.82; 95% CI (0.58, 5.73); *P* = 0.31] (Fig. 3E).

Lastly, we performed subgroup analysis based on the type of biologic augmentation: (1) BMAC group: 32.8% (66/201) of femoral heads in the stem cell group collapsed, whereas the proportion in the control group was 65.3% (115/176). The pooled data indicated that it was not statistically significant [OR = 2.78; 95% CI (0.64, 12.13); *P* = 0.17] (Fig. 3F). (2) BMMSCs/BMMSs/PBSCs group: 35/136 (25.7%) hips in the stem cell group and 47/107 (43.9%) in the control group were observed with collapse of the femoral head. It was statistically significant [OR = 3.30; 95% CI (1.74, 6.25); *P* = 0.0002] (Fig. 3G).

##### Radiological progression

Failure of intervention was defined as the radiological progression of necrotic zone, so this outcome was extracted from 11 [10, 23, 24, 26, 29–34, 39] studies.

**Fig. 4:**
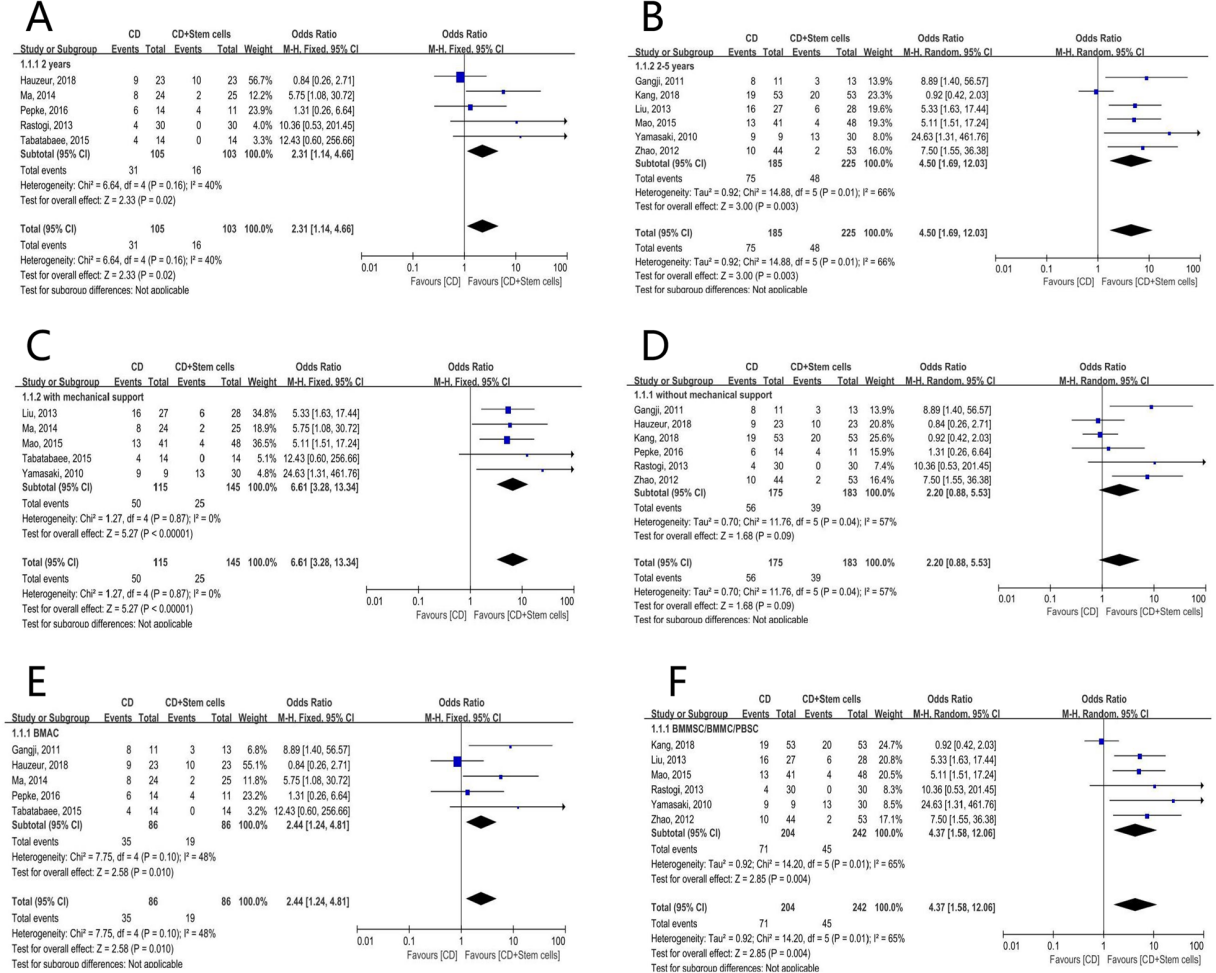
Forest plots of the rate of radiographic progression. Subgroup analysis according to **A** the duration of follow-up in 2 years, **B** the duration of follow-up in 2–5 years, **C** with structural support, **D** without structural support, **E** stem cell therapy of the BMAC group, **F** stem cell therapy of the BMMSCs/BMMSs/PBSCs group. (BMAC bone marrow aspirate concentrate, BMMCs bone marrow mononuclear cells, BMMSCs bone marrow mesenchymal stem cells, CD core decompression, CI confidence interval, df degree of freedom, M-H Mantel–Haenszel test, PBSCs peripheral blood stem cells)

First of all, subgroup analysis was based on the follow-up duration. (1) 2 year-follow-up: 15.5% (16/103) of femoral heads in the stem cell group and 29.5% (31/105) in the control group progressed radiologically. It was not statistically significant [OR = 2.31; 95% CI (1.14, 4.66); *P* = 0.02] (Fig. 4A). (2) 2 to 5 year-follow-up: 48/225 (21.3%) hips in the stem cell group and 75/185 (40.5%) in the control group progressed. The pooled data were statistically significant [OR = 4.50; 95% CI (1.69, 12.03); *P* = 0.003] (Fig. 4B).

In the subgroup analysis was based on whether subchondral mechanical support was performed, we discovered that (1) in CD with mechanical support group, 25/145 (17.2%) and 50/115 (43.5%) hips experienced radiological progressions in stem cell group and control group [OR = 6.61; 95% CI (3.28, 13.34); *P* < 0.00001] (Fig. 4C), and (2) in CD without mechanical support group, (39/183) (21.3%) and (56/175) (32.0%) hips experienced radiological progressions in stem cell group and control group [OR = 2.20; 95% CI (0.88, 5.53); *P* = 0.09] (Fig. 4D).

We thirdly performed subgroup analysis according to the type of cell therapy: (1) BMAC group: 22.1% (19/86) of femoral heads in the stem cell group progressed, while in the control group the figure was 35/86 (40.7%) [OR = 2.44; 95% CI (1.24, 4.81); *P* = 0.01] (Fig. 4E). (2) BMMSCs/BMMSs/PBSCs group: 45/242 hips (18.6%) in the stem cell group and 71/204 (34.8%) in the control group were observed with radiological progression. The pooled data indicated a statistically significance [OR = 4.37; 95% CI (1.58, 12.06); *P* = 0.004] (Fig. 4F).

##### Conversion to THA

THA is the ultimate surgery for those failed hip-preserving cases. And therefore, conversion to THA is a crucial outcome of these studies and this was documented in 17 [10, 23–27, 29–39] of the included studies.

**Fig. 5:**
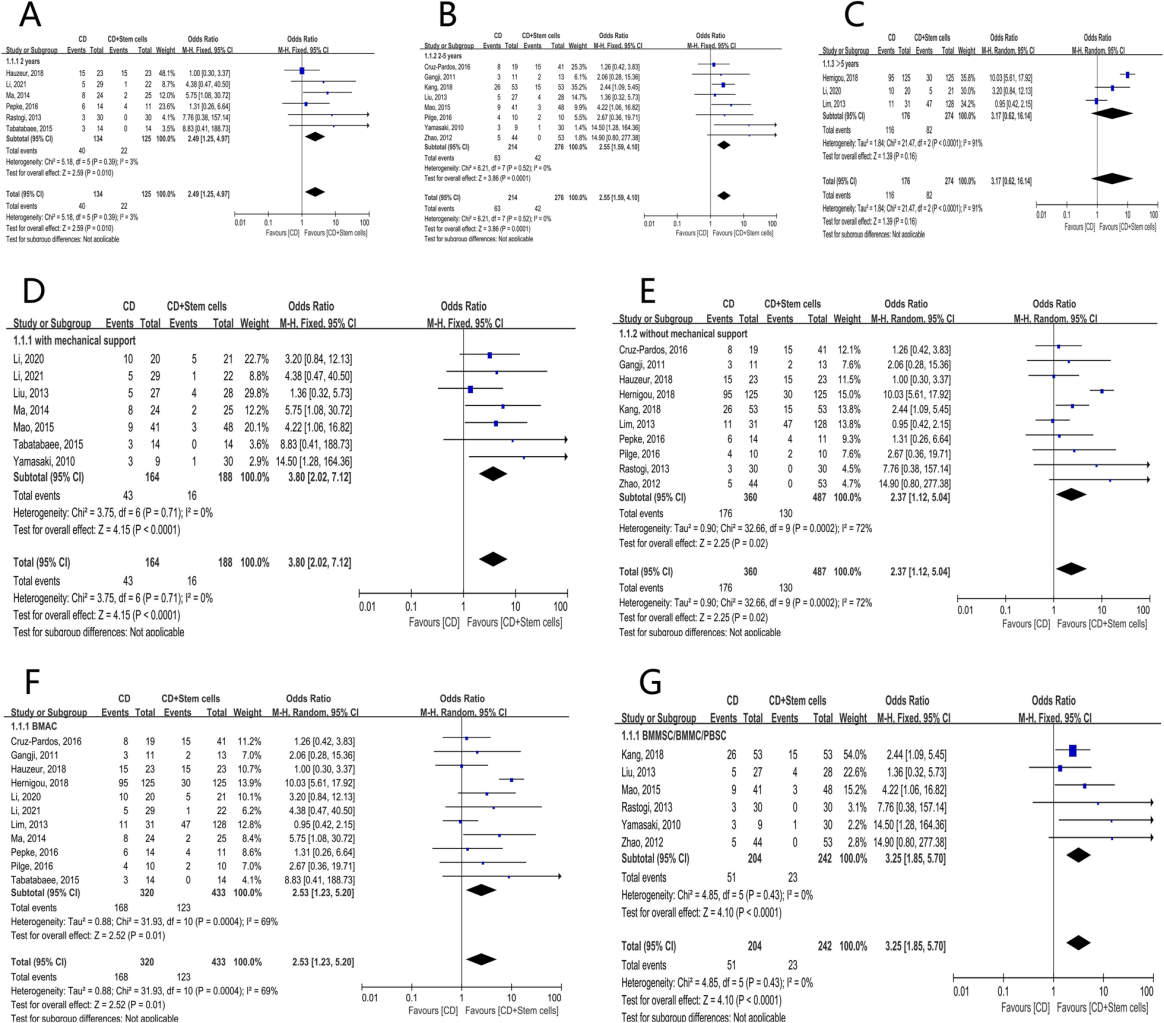
Forest plots of the rate of THA conversion. Subgroup analysis according to **A** the duration of follow-up in 2 years, **B** the duration of follow-up in 2–5 years, **C** the duration of follow-up longer than 5 years, **D** with structural support, **E** without structural support, **F** stem cell therapy of the BMAC group, **G** stem cell therapy of the BMMSCs/BMMSs/PBSCs group. (BMAC bone marrow aspirate concentrate, BMMCs bone marrow mononuclear cells, BMMSCs bone marrow mesenchymal stem cells, CD core decompression, CI confidence interval, df degree of freedom, M–H Mantel–Haenszel test, PBSCs peripheral blood stem cells)

In the subgroup analysis based on the follow-up duration, (1) 22/125 (17.6%) hips in stem cell group and 40/134 (30.0%) hips in control group received THA during 2 years’ follow-up. The pooled results revealed a significant difference between the groups [OR = 1.69; 95% CI (1.13, 2.51); *P* = 0.01] (Fig. 5A). (2) When the follow-up duration lasted to 5 years, 42/276 (15.2%) hips in stem cell group and 63/214 (29.4%) hips in control group received THA. It was statistically significant [OR = 1.94; 95% CI (1.38, 2.71); *P* = 0.0001] (Fig. 5B). However, (3) for the data of follow-up longer than 5 years, 82/274 (29.9%) and 116/176 (65.9%) hips were conversed to THA, respectively, and the pooled data did not indicate statistically significance [OR = 3.17; 95% CI (0.62, 16.14); *P* = 0.16] (Fig. 5C).

Secondly, subgroup analysis was based on the utilization of subchondral mechanical support. (1) CD with mechanical support: 8.5% (16/188) of femoral heads in the stem cell group and 26.2% (43/164) in the control group conversed to THA ultimately. It was statistically significant [OR = 3.80; 95% CI (2.02, 7.12); *P* < 0.0001] (Fig. 5D). (2) CD without mechanical support: 130/487 hips (26.7%) in the stem cell group and 176/360 (48.9%) in the control group had THA terminally. The pooled data were statistically significant [OR = 2.37; 95% CI (1.12, 5.04); *P* = 0.02] (Fig. 5E).

When examining the kind of cell therapy, we discovered the pooled resulted favored stem cell group in both BMAC (SG: 123/433, 28.4% vs CG: 168/320, 52.5%; OR = 2.53; 95% CI (1.23, 5.20); *P* = 0.01) (Fig. 5F) and BMMSCs/BMMSs/PBSCs subgroup (SG: 23/242, 9.5% vs CG: 51/204, 25.0%; OR = 3.25; 95% CI (1.85, 5.70); *P* < 0.0001) (Fig. 5G).

##### Publication bias

Egger test was carried out for all the forest plots which included more than 2 studies, and the results are shown in Table 4. For the rate of radiographic progression of 2-to-5-year follow-up, using subchondral bone graft and specific stem cells transplantation, P of Egger test was 0.001, 0.016 and 0.025, respectively, and therefore, possible publication bias was suggested. Funnel plots of standard error by effect size was illustrated in (Additional file 1: Fig. S1, S2, S3).

**Table 4:** Publication bias evaluated by egger test

Subgroup	Outcomes	Number of trails	*P* (Egger test)
Follow-up (2 years)	Collapse of the femoral head	2	–
	Radiological progression	5	0.411
	Conversion to THA	6	0.325
Follow-up (2–5 years)	Collapse of the femoral head	5	0.521
	Radiological progression	6	0.001
	Conversion to THA	8	0.423
Follow-up (> 5 years)	Collapse of the femoral head	1	–
	Conversion to THA	3	–
Subchondral (with mechanical support)	Collapse of the femoral head	4	0.442
	Radiological progression	5	0.016
	Conversion to THA	7	0.295
Subchondral (without mechanical support)	Collapse of the femoral head	4	0.312
	Radiological progression	6	0.665
	Conversion to THA	10	0.383
Type of stem cells (BMAC)	Collapse of the femoral head	4	0.768
	Radiological progression	5	0.542
	Conversion to THA	11	0.288
Type of stem cells (BMMSCs/BMMSs/PBSCs)	Collapse of the femoral head	4	0.434
	Radiological progression	6	0.025
	Conversion to THA	6	0.357

BMAC bone marrow aspirate concentrate, BMMCs bone marrow mononuclear cells, BMMSCs bone marrow mesenchymal stem cells, PBSCs peripheral blood stem cells, THA total hip arthroplasty

##### Assessment of the quality of recommendations

The GRADE system was used to evaluate all subgroups’ results of the three main outcomes in the present study. For the outcome of femoral head collapse, very low-quality evidence was found in the subgroups of 2-year follow-up, non-mechanical support and BMAC, while the quality was rated as low in the subgroups of 2–5-year follow-up, longer than 5-year follow-up, with mechanical support, and BMMSCs/BMMSs/PBSCs. For the outcome of radiological progression, very low-quality evidence was found in the subgroups of 2–5-year follow-up, non-mechanical support and BMMSCs/BMMSs/PBSCs, while moderate quality evidence was found in the subgroups of 2-year follow-up and with mechanical support, and high-quality evidence was found in the BMAC subgroup. For the outcome of conversion to THA, very low-quality evidence was found in the subgroups of longer than 5-year follow-up, non-mechanical support and BMAC, while low-quality evidence was found in the subgroups of 2–5-year follow-up, mechanical support, and BMMSCs/BMMSs/PBSCs (Table 5).

**Table 5:** Assessment of the quality of recommendations

Subgroup	Outcomes	Number of trails	Number of patients			Follow-up (Y)	Relative effect (95% CI)	Quality of the evidence (GRADE)
			SG		CG			
Follow-up (2 years)	Collapse of the femoral head	2	12/35		16/32	2	OR 4.5 (0.07 to 307.07)	⊕ ⊝ ⊝ ⊝ very low
	Radiological progression	5	16/103		31/105	2	OR 2.49 (0.85 to 7.32)	⊕ ⊕ ⊕ ⊝ moderate
	Conversion to THA	6	22/125		40/134	2	OR 2.29 (1.07 to 4.88)	⊕ ⊕ ⊕ ⊕ high
Follow-up (2–5 years)	Collapse of the femoral head	5	54/177		56/126	3.2	OR 2.41 (1.09 to 5.32)	⊕ ⊕ ⊝ ⊝ low
	Radiological progression	6	48/225		75/185	4.0	OR 4.5 (1.69 to 12.03)	⊕ ⊝ ⊝ ⊝ very low
	Conversion to THA	8	42/276		63/214	3.5	OR 2.42 (1.48 to 3.95)	⊕ ⊕ ⊝ ⊝ low
Follow-up (> 5 years)	Collapse of the femoral head	1	35/125		90/125	25	OR 6.61 (3.81 to 11.49)	⊕ ⊕ ⊝ ⊝ low
	Conversion to THA	3	82/274		116/176	14.1	OR 3.17 (0.62 to 16.14)	⊕ ⊝ ⊝ ⊝ very low
Subchondral (with mechanical support)	Collapse of the femoral head	4	22/118		36/86	2.4	OR 6.15 (1.91 to 19.74)	⊕ ⊕ ⊝ ⊝ low
	Radiological progression	5	25/145		50/115	2.3	OR 6.11 (3 to 12.46)	⊕ ⊕ ⊕ ⊝ moderate
	Conversion to THA	7	16/188		43/164	3.4	OR 3.73 (1.96 to 7.11)	⊕ ⊕ ⊝ ⊝ low
Subchondral (without mechanical support)	Collapse of the femoral head	4	79/219		126/197	3.2	OR 1.82 (0.58 to 5.73)	⊕ ⊝ ⊝ ⊝ very low
	Radiological progression	6	39/183		56/175	3.4	OR 2.2 (0.88 to 5.53)	⊕ ⊝ ⊝ ⊝ very low
	Conversion to THA	10	130/487		176/360	5.9	OR 2.37 (1.12 to 5.04)	⊕ ⊝ ⊝ ⊝ very low
Type of stem cells (BMAC)	Collapse of the femoral head	4	66/201		115/176	8.2	OR 2.78 (0.64 to 12.13)	⊕ ⊝ ⊝ ⊝ very low
	Radiological progression	5	19/86		35/86	2.6	OR 2.82 (0.96 to 8.3)	⊕ ⊕ ⊕ ⊕ high
	Conversion to THA	11	123/433		168/320	5.8	OR 2.53 (1.23 to 5.2)	⊕ ⊝ ⊝ ⊝ very low
Type of stem cells (BMMSCs/BMMSs/PBSCs)	Collapse of the femoral head	4	35/136		47/107	3.0	OR 3.92 (1.17 to 13.14)	⊕ ⊕ ⊝ ⊝ low
	Radiological progression	6	45/242		71/204	3.2	OR 4.02 (0.88 to 18.29)	⊕ ⊝ ⊝ ⊝ very low
	Conversion to THA	6	23/242		51/204	3.2	OR 2.66 (1.28 to 5.5)	⊕ ⊕ ⊝ ⊝ low

CI confidence interval, OR Odds Ratio, BMAC bone marrow aspirate concentrate, BMMCs bone marrow mononuclear cells, BMMSCs bone marrow mesenchymal stem cells, PBSCs peripheral blood stem cells, THA total hip arthroplasty, GRADE Grading of Recommendations Assessment, Development and Evaluation

## Discussion

Stem cell therapy combined with core decompression, as utilized in more than 20 clinical studies [40], was a prevalent hip-preserving strategy to treat ANFH. Nonetheless, the efficacy of stem cell therapy was inconclusive based on the current literature [41]. We included the level III to level I evidence in the present systematic review and meta-analysis with the purpose to analyze the outcome after stem cell therapy in a comprehensive way by further subgroup analysis. Overall, our results supported our initial hypothesis that the hypothesis was that: (1) the augmentation using cell therapy would postpone the progression of ANFH and reduce the conversion rate of THA. (2) The mechanical support of the subchondral bone in the femoral head would be advantageous. (3) The outcomes would not vary while using either MSCs or bone marrow aspirate concentrate (BMAC). Additionally, in the subgroup analysis, we found that stem cell therapy could lowering the rate of collapse, radiographic progression and THA conversion on a mid-term basis. We also confirmed the necessity of subchondral mechanical support after CD for its advantage in avoiding collapse and disease progression. Thirdly, the utilization of a specific type of stem cell was indicated to be more efficient than BMAC.

Core decompression is a hip-preserving surgical technique that aims to mitigate edema and improve circulation of the femoral head by decreasing intraosseous pressure, and thus, it has the potential to prevent or postpone THA [42]. Conversely, the clinical results of CD alone were still controversial in the current literature because of the inconclusive success rate especially for cases of collapse stage [43]. Mont [44] reported a success of only 47% in ARCO stage III cases. Similarly, Song [45] reported the survival rate of Ficat stage I, II and III was 79%, 77% and 35%, respectively, in a study with a minimum 5-year follow-up. These unsatisfying results could be attributed to large diameter core decompression, deprivation of regional MSCs, inaccurate or incomplete bone graft compaction or early postoperative weight-bearing [24].

In recent decades, enthusiasm has been aroused for applying osteogenic precursors to necrotic lesions in ANFH for their capacity to differentiate to diverse cell lineages. The scientific foundation underlying stem cell therapy is to provide osteoprogenitor and vascular progenitor cells to facilitate bone remodeling and repair in the necrotic area [46]. To add to that, strategies that stimulate and enhance the mobilization and homing capacity of MSCs also attracted growing interests [47]. Individual studies of stem cell therapy combined with CD revealed promising results. Gangji [30] claimed that the strategy of stem cell application afforded a significant improved hip function, reduced volume of necrotic lesions, and delayed radiographic progression. 25-year study conducted by Hernigou [35] indicated that stem cell therapy reduced collapse and THA conversion rate while comparing with CD alone. Our previous 10-year result also favored the employment of stem cell since it provided better subjective assessment scores and longer average survival time[37]. These were in accordance with the results of our meta-analysis that stem cell augmentation plus CD reduced the collapse rate by 2.97 times, the radiographic progression rate by 3.52 times and THA conversion rate by 2.85 times compared with CD alone. Nan et al. had demonstrated that resveratrol (Res) can potentially reverse abnormal osteogenesis during ANFH by suppressing osteoclastogenesis via modulating levels of sirtuin1 (Sirt1), nuclear transcription factor-κB (NF-κB), and receptor activator of NF-κB ligand (RANKL) [48]. Zhang et al. found that during treatment of ANFH with BMSCs, the transplanted cells underwent significant stress-induced apoptosis and senescence in the oxidative stress microenvironment of the necrotic area, significantly limiting their efficacy. Subsequent studies by the authors revealed that upregulation of Parkin and downregulation of P53 in BMSCs effectively counteracted stress-induced apoptosis and senescence and improved the therapeutic effect of BMSC transplantation in early steroid-induced ANFH [49]. All of these findings provided new avenues for the subsequent treatment of ANFH.

Nonetheless, owing to the lack of a standardization in the regard of the qualitative and quantitative guidelines of the harvest methods, processing and transplantation of cells, there was a dramatic heterogeneity in the current published studies. Various mesenchymal cells were applied in the hip-preserving procedures, including BMMSCs, BMMCs, PBSCs, human umbilical cord mesenchymal stem cells, etc. [50, 51]. These cells would promote the secretion of osteogenic and angiogenic factors in the necrotic area [52]. Aside from those, BMAC, proposed by Hernigou [53] firstly, was preferred in numerous studies because of its convenience in harvesting and processing. BMAC is indicated to provide higher concentration of chondrogenic, affirmative stromal cells, lymphocytes, neutrophils, monocytes, and platelets in various stages of differentiation [54, 55]. However, it should be noticed that the number of cells in BMAC range from 90,000 to 3.46 × 10^9^ and the transplanted volume varied from 1 to 60 ml. Moreover, most of the cells in BMAC are not mesenchymal or vascular progenitor cells [3]. We suspected that it might attributed to the ambiguous results of CD plus BMAC therapy with respect of collapse rate and progression rate while comparing with CD alone in our pooled data.

With the concern of subchondral structural weakness after CD, mechanical augmentation is another fundamental approach in the treatment of ANFH [56]. There are various choices to enhance the mechanical support, including vascularized fibula grafts, autologous cancellous bone grafts, allografts and porous tantalum, etc. [57, 58]. Chen [59] reported the optimizing mid- and long-term results of bone graft impaction through the CD track, especially for those early pre-collapse cases. In addition to structural enhancement, bone grafts also provide a microenvironment for bone remodeling and angiogenesis [60]. Dou [61] founded that porous tantalum could promote the proliferation and adhesion of BMSCs via activation of the MAPK/ERK pathway, so that it could up-regulate the expression of osteogenic genes and promote the osteogenic differentiation of BMSCs in vitro. Our pooled data also supported mechanical enhancing procedures for lowering the risk of disease progression. It is worthy noticing the utilization of synthesized and bio-inductive material. Liu [24] proposed a 10-mm single drilling technique in combination with granular porous medical nano-hydroxyapatite / polyamide 66 composite bone filling material transplantation and reported promising clinical results. Previously, our center introduced angioconductive bioceramic rod grafting combined with BMAC to treat early-stage cases and reported satisfying results of improved hip function and a higher survivorship as well [38].

Our pooled data in subgroup analysis based on the duration of follow-up favored the use of stem cell therapy, which is in accordance with the current literature [62]. Conversely, the efficacy of stem cell augmentation was inconclusive on a short-term basis, which was consistent with the systematic review of Andronic [41]. For the studies with a follow-up of 2 years, Hauzeur [10] only included the cases of ARCO stage III and more than a half with the etiology of corticosteroid-use. Similarly, corticosteroid-use and idiopathic factor took high proportion for the risk factor of patients included in Rastogi’s study [39]. As was revealed in the current literature, corticosteroid would influence the treatment outcome because MSCs in these patients not only had impaired activity but also tend to differentiate into adipose cells instead of osteoblasts, by imposing adverse effects on bone matrix, cell apoptosis, lipid metabolism and angiogenesis [63–65]. Therefore, this etiology was considered a negative prognostic factor for hip preservation. In regard of the long-term results, although Hernigou [35] reported a lower collapse rate and THA conversion rate, it was not consistent with Li [37] and Lim [27]. Future studies with follow-up longer than 5 years and larger sample sizes may provide more persuasive evidence.

This systematic review and meta-analysis had some limitations. First, due to the limited RCTs, we included 7 retrospective studies with good quality. Despite of this, the enrolled numbers of patients of hips was still small. Therefore, it needs large sample size, multi-center, prospective, randomized controlled studies to test and verify this inference. Second, although we performed subgroup analysis to balance the heterogeneity of follow-up duration, surgical technique and type of cell therapy, the approach of bone grafting and the numbers of cells in the treatment were still diverse. Thirdly, all the included studies reported the positive outcomes of stem cell therapy, which might introduce publication bias. Fourth, the included studies involved various types of scoring system and the data were reported in different forms, and thus, we did not extract and synthesize the quantitative data. Additionally, although two investigators reviewed the results and data based on the standardized form and came to an agreement, search bias and extractor bias may still have occurred. Last but not least, we only included studies published in English which would lead to language bias.

## Conclusion

Stem cell therapy combined with core decompression was more effective in preventing collapse, radiographic progression and conversion to THA.

## Supplementary Information

**Additional file 1: Figure S1:** Funnel plots of the rate of collapse. Subgroup analysis according to (A) the duration of follow-up in 2 years, (B) the duration of follow-up in 2–5 years, (C) with structural support, (D) without structural support, (E) stem cell therapy of the BMAC group, (F) stem cell therapy of the BMMSCs/BMMSs/PBSCs group. (BMAC bone marrow aspirate concentrate, BMMCs bone marrow mononuclear cells, BMMSCs bone marrow mesenchymal stem cells, PBSCs peripheral blood stem cells).

**Additional file 2: Figure S2:** Funnel plots of the rate of radiographic progression. Subgroup analysis according to (A) the duration of follow-up in 2 years, (B) the duration of follow-up in 2–5 years, (C) with structural support, (D) without structural support, (E) stem cell therapy of the BMAC group, (F) stem cell therapy of the BMMSCs/BMMSs/PBSCs group. (BMAC bone marrow aspirate concentrate, BMMCs bone marrow mononuclear cells, BMMSCs bone marrow mesenchymal stem cells, PBSCs peripheral blood stem cells).

**Additional file 3: Figure S3:** Funnel plots of the rate of radiographic progression. Subgroup analysis according to (A) the duration of follow-up in 2 years, (B) the duration of follow-up in 2–5 years, (C) the duration of follow-up longer than 5 years, (D) with structural support, (E) without structural support, (F) stem cell therapy of the BMAC group, (G) stem cell therapy of the BMMSCs/BMMSs/PBSCs group. (BMAC bone marrow aspirate concentrate, BMMCs bone marrow mononuclear cells, BMMSCs bone marrow mesenchymal stem cells, PBSCs peripheral blood stem cells). (PDF 1870 kb)

## Data Availability

The data and materials used and/or analyzed during the current study are not publicly available but available from the corresponding author on reasonable request.

## Abbreviations

ANFH: Avascular necrosis of the femoral head
ARCO: Association Research Circulation Osseous
BMAC: Bone marrow aspirate concentrate
BMMCs: Bone marrow mononuclear cells
BMMSCs: Bone marrow mesenchymal stem cells
CD: Core decompression
CG: Control group
GRADE: Grading of Recommendations Assessment, Development and Evaluation
HHS: Harris hip score
JOA: Japanese Orthopaedic Association
LoE: Level of evidence
MAP: Merle D’Aubigné and Postel score
ANFH: Avascular necrosis of the femoral head
OR: Odd Ratio
PBSCs: Peripheral blood stem cells
SG: Stem cell group
VAS: Visual analogue scale
WOMAC: Western Ontario and McMaster Universities Arthritis Index osteoarthritis scoring
