# Supplementary figures and images for: Stem cell therapy combined with core decompression versus core decompression alone in the treatment of avascular necrosis of the femoral head: a systematic review and meta-analysis
Source: J Orthop Surg Res. 2023 Aug 2;18:560. doi: 10.1186/s13018-023-04025-8 (PMC10398910; doi:10.1186/s13018-023-04025-8)

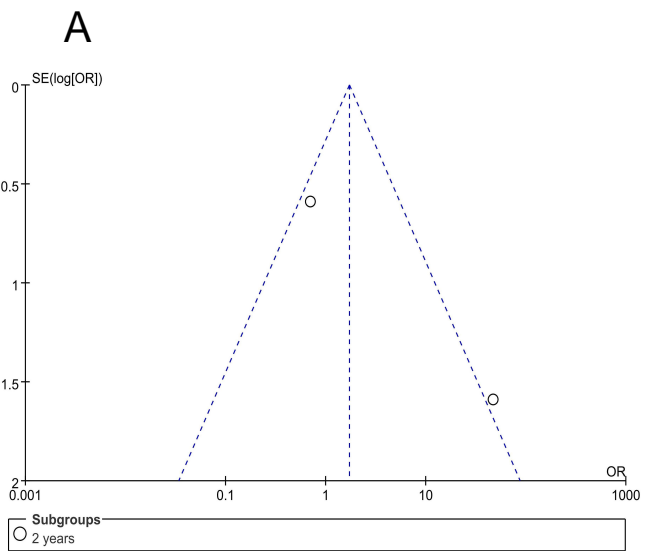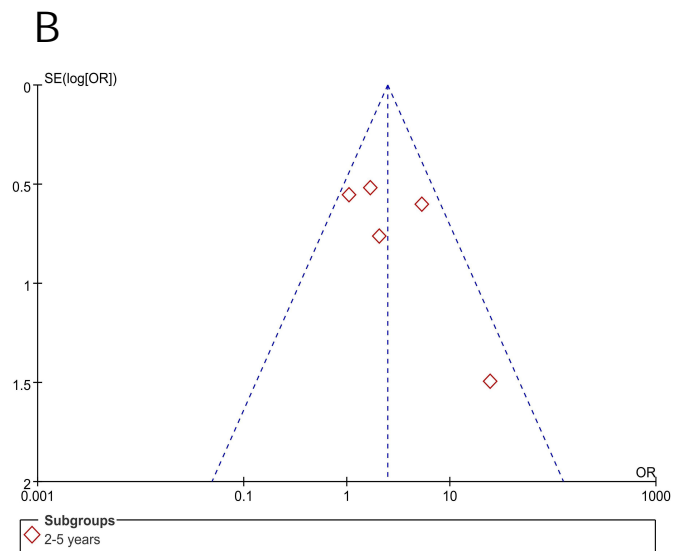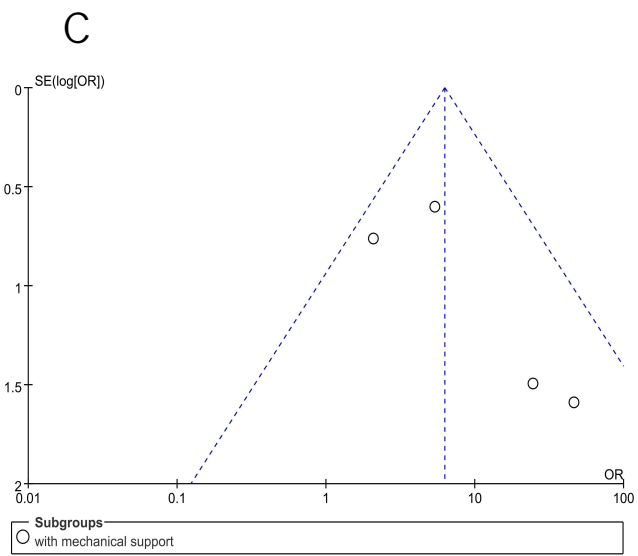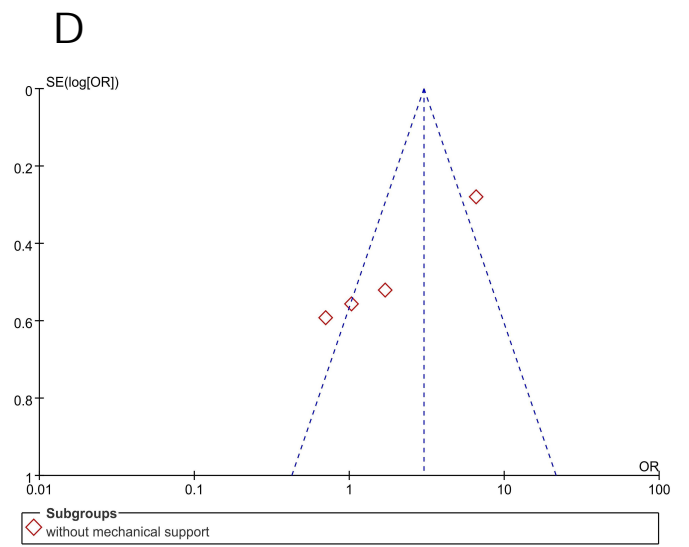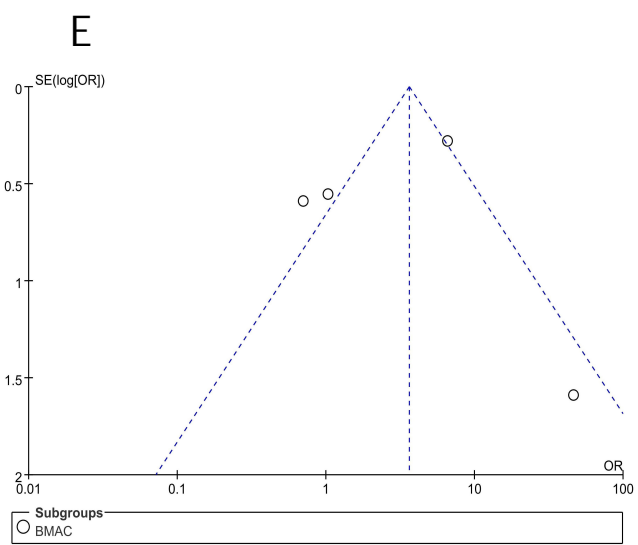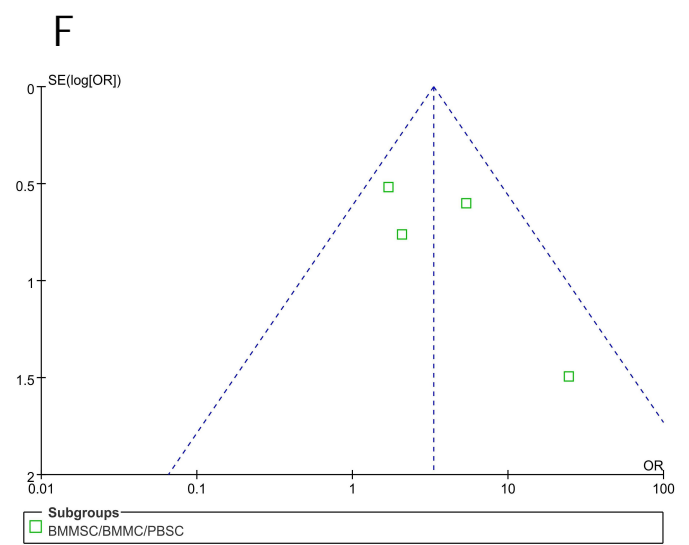

Supplement: Supplementary file 1 — Additional file 1: Figure S1: Funnel plots of the rate of collapse. Subgroup analysis according to (A) the duration of follow-up in 2 years, (B) the duration of follow-up in 2–5 years, (C) with structural support, (D) without structural support, (E) stem cell therapy of the BMAC group, (F) stem cell therapy of the BMMSCs/BMMSs/PBSCs group. (BMAC bone marrow aspirate concentrate, BMMCs bone marrow mononuclear cells, BMMSCs bone marrow mesenchymal stem cells, PBSCs peripheral blood stem cells). [file 13018_2023_4025_MOESM1_ESM.pdf]

# A

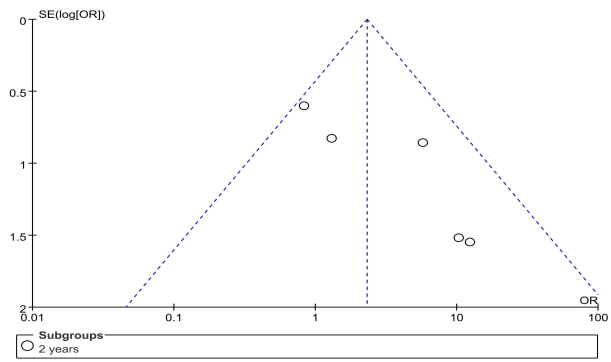

# B

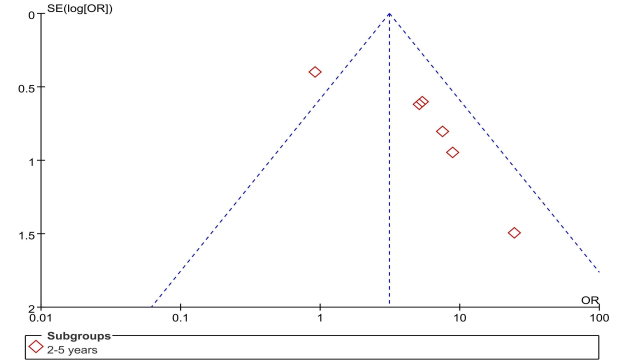

# C

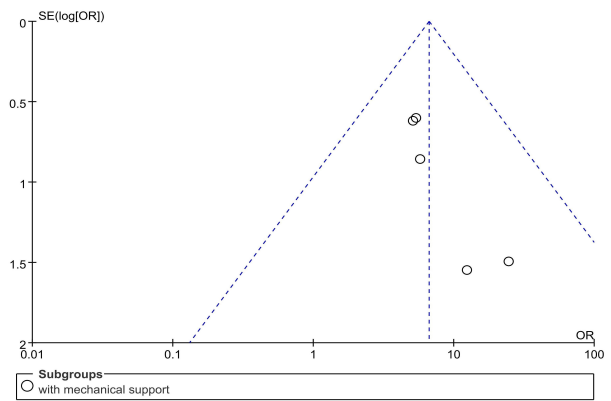

# D

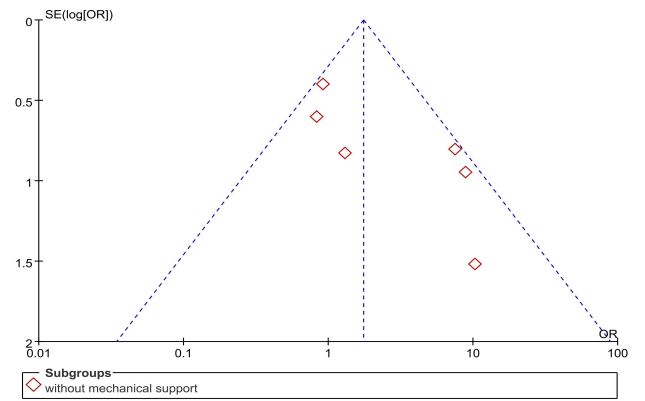

# E

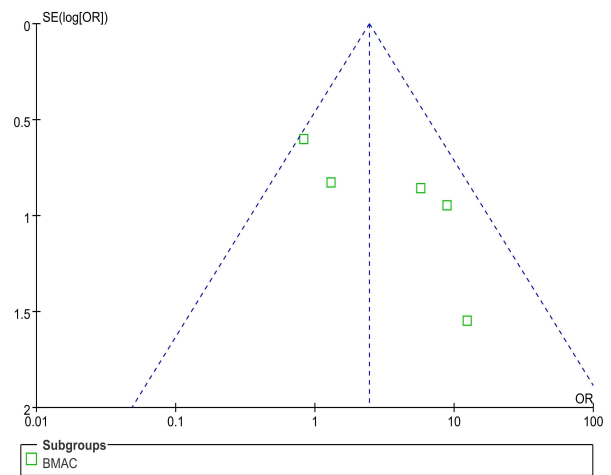

# F

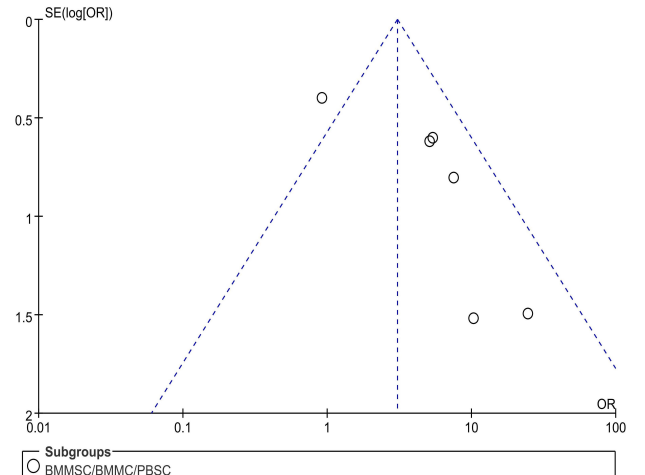

Supplement: Supplementary file 2 — Additional file 2: Figure S2: Funnel plots of the rate of radiographic progression. Subgroup analysis according to (A) the duration of follow-up in 2 years, (B) the duration of follow-up in 2–5 years, (C) with structural support, (D) without structural support, (E) stem cell therapy of the BMAC group, (F) stem cell therapy of the BMMSCs/BMMSs/PBSCs group. (BMAC bone marrow aspirate concentrate, BMMCs bone marrow mononuclear cells, BMMSCs bone marrow mesenchymal stem cells, PBSCs peripheral blood stem cells). [file 13018_2023_4025_MOESM2_ESM.pdf]

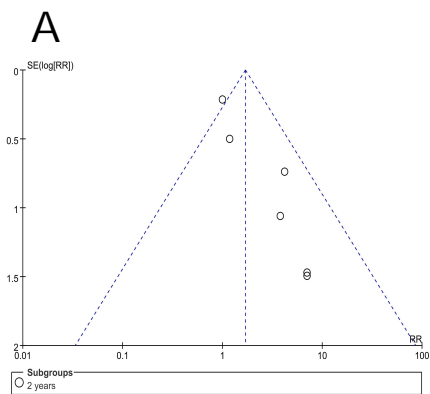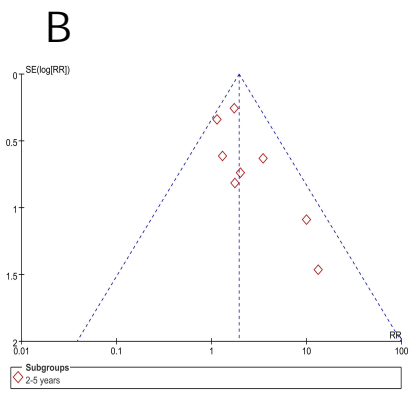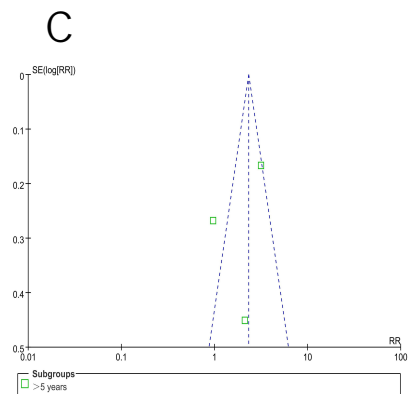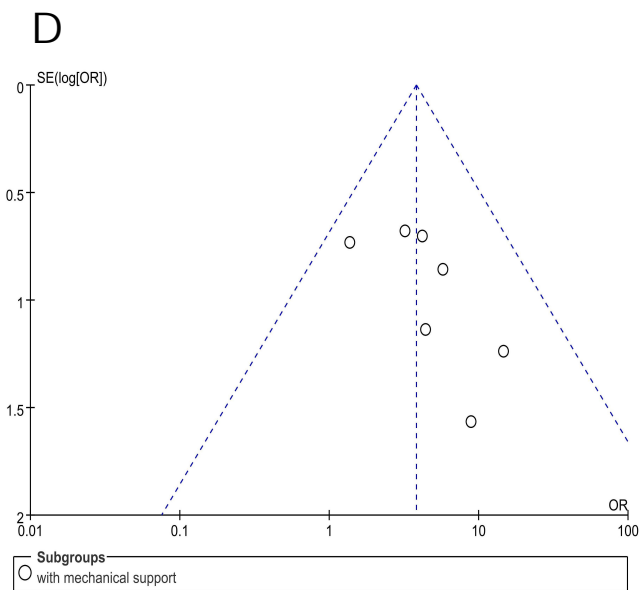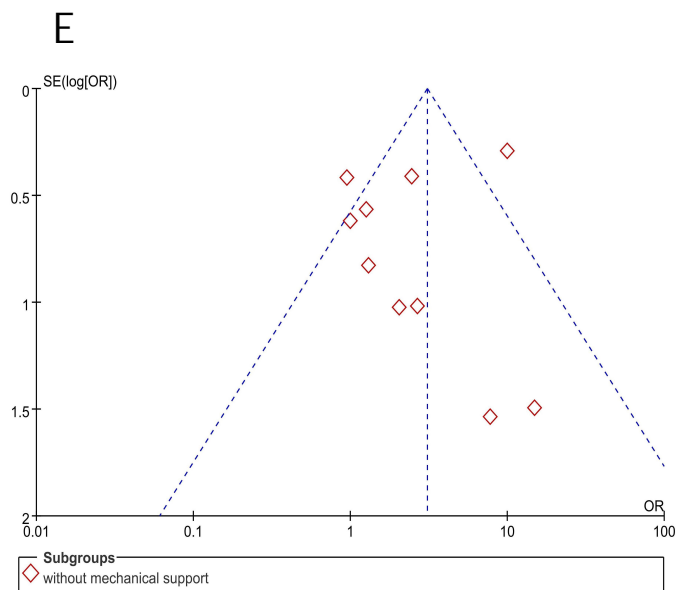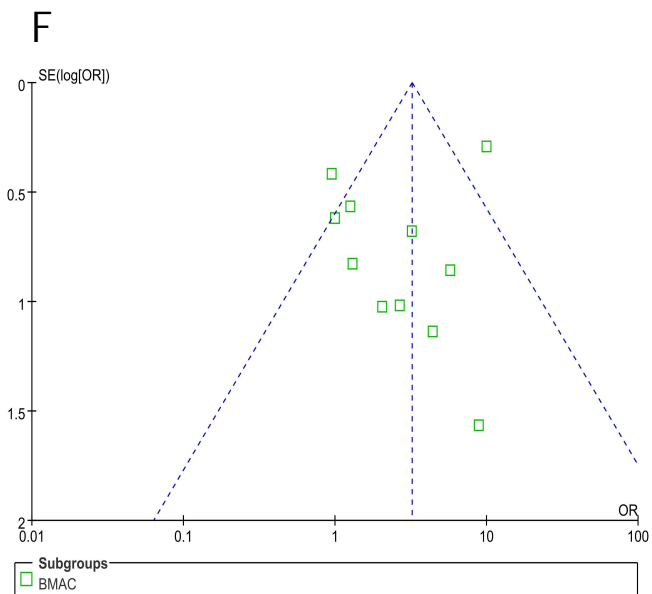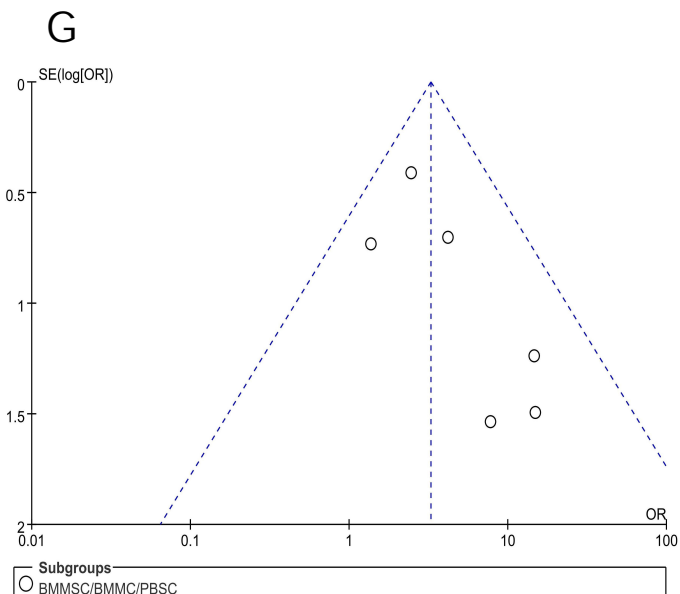

Supplement: Supplementary file 3 — Additional file 3: Figure S3: Funnel plots of the rate of radiographic progression. Subgroup analysis according to (A) the duration of follow-up in 2 years, (B) the duration of follow-up in 2–5 years, (C) the duration of follow-up longer than 5 years, (D) with structural support, (E) without structural support, (F) stem cell therapy of the BMAC group, (G) stem cell therapy of the BMMSCs/BMMSs/PBSCs group. (BMAC bone marrow aspirate concentrate, BMMCs bone marrow mononuclear cells, BMMSCs bone marrow mesenchymal stem cells, PBSCs peripheral blood stem cells). (PDF 1870 kb) [file 13018_2023_4025_MOESM3_ESM.pdf]
